# Graduate-level medical doctors in Uganda have limited awareness of warning, specific signs, and immunological investigations of primary immunodeficiencies

**DOI:** 10.3389/fimmu.2026.1766520

**Published:** 2026-03-26

**Authors:** Obondo James Sande, Brian Matovu, Tracy Kongai, Joseph Olobo

**Affiliations:** 1 Department of Immunology and Molecular Biology, School of Biomedical Sciences, College of Health Sciences, Makerere University, Kampala, Uganda; 2 Department of Genetics and Genome Sciences, Case Western Reserve University School of Medicine, Cleveland, OH, United States; 3 Department of Microbiology and Immunology, Faculty of Medicine, Lira University, Lira, Uganda

**Keywords:** clinical immunology knowledge, clinical manifestations, IEIs warning and specific signs, immunological investigations, primary immunodeficiency

## Abstract

**Background:**

Primary Immunodeficiencies are inborn errors of immunity (IEIs) characterized by impaired immune function, resulting in recurrent infections, inflammation, autoimmunity, atopy, and malignancies. Although significant advances in diagnosis have been made in high-income countries, IEIs remain underdiagnosed in resource-limited settings like Uganda, where infectious diseases dominate clinical attention. Recognizing IEIs requires strong foundational knowledge of immune system functions, clinical manifestations, and relevant immunological investigations. This study assessed Ugandan doctors’ knowledge in these areas.

**Method:**

A cross-sectional quantitative study was conducted among 114 doctors pursuing Master’s degrees in Pediatrics, Internal Medicine, Immunology, and Clinical Microbiology at Makerere University. A 42-item questionnaire assessed knowledge across four domains: immune cell functions and clinical manifestations of IEIs-related defects, warning signs in adults and children, specific IEIs signs, and relevant immunological investigations. Responses were “True” (correct) or “Not sure” (incorrect/uncertain), and proportions of correct answers were calculated.

**Results:**

Respondents were mostly female (56.16%). Knowledge of immune cell functions and related clinical manifestations was high, with 77–97% correct responses. Awareness of key warning signs varied; while many recognized poor growth (76%) and family history (72%) as potential indicators, most scored low (13–44%) on other warning signs and specific IEIs features. Knowledge of immunological investigations was low: over 90% reported no experience with essential immunological tests, even basic investigations, such as CBC interpretation for IEIs screening were reported by only 6%.

**Conclusion:**

Ugandan doctors show strong foundational immunology knowledge but limited awareness of warning and specific IEIs signs, and almost no experience with diagnostic immunological tests.

## Introduction

Inborn errors of Immunity (IEIs) are a diverse and heterogeneous group of genetic disorders characterized by poor or absent immune system function, which predisposes affected individuals to recurrent infections, inflammation, autoimmunity, atopy, and malignancies (1). There are approximately 500 genetically defined IEIs, and although many may be rare, there is often a delay in diagnosis (1). While there have been many advances in research, diagnosis and management of IEIs in affluent countries, they remain underdiagnosed or missed altogether in resource-limited settings like Uganda, where the primary focus is on diagnosing infections (2, 3). However, IEIs influence the occurrence and severity of infectious diseases (3). This could be attributed to a lack of awareness among medical professionals about IEIs, regarding the basic functions of the different parts of the immune system, the clinical manifestations related to defects in various immune system components, important IEIs -focused immunological investigations, and the necessary infrastructure for IEIs -focused laboratory testing for early diagnosis.

Recent studies show that fundamental knowledge of the basic functions of the immune cells and clinical manifestations related to defects in the different immune cell pathways improves doctors’ understanding of how recurrent infections, autoimmunity, atopy, malignancies, and other disorders relate to IEIs, ultimately improving awareness, early diagnosis, and treatment of IEIs (4–6). Thus, when it comes to clinical manifestations and immunological investigations of patients with suspected IEIs, there is a need to go back to the basic functions of the different parts of the immune system: B cells make antibodies and have a role in opsonization and complement activation. T cells are important in viral immunity and immunity to intracellular pathogens and have an immune regulatory role. Phagocytes clear microbes and play a role in antigen presentation. On the other hand, natural killer cells destroy virally infected cells and tumor cells (7–10).

Based on the knowledge of the fundamental functions of parts of the immune system, physicians, pediatricians, and clinical immunologists can understand how defects in individual immune pathways lead to specific symptoms and signs. For example, B cell defects cause bacterial infections with predominantly capsulated microorganisms such as *H. influenzae* and *Strepcoccus pneumoniae* (11, 12). T cell defects lead to opportunistic infections with mainly intracellular pathogens, and autoimmunity, while phagocyte (neutrophil) defects result in bacterial infections and poor wound healing; complement defects cause bacterial infections and autoimmunity (13). On the other hand, natural killer cell deficiency leads to increased susceptibility to viral infections and malignancy (13, 14). The clinical manifestations of IEIs are well-summarized by several studies, including the ten warning signs developed by the Jeffrey Modell Foundation in 1993 (15). A thorough history of infections, including infection sites, severity, recurrent infections with unusual organisms, and responses to treatment, is essential. Additionally, particular attention should be paid to family history, which is recognized as one of the strongest predictors of IEIs (15, 16).

Depending on the clinical presentation, clinicians can appropriately guide immunological investigations. There are assays available to examine different pathways. The first is to quantify individual immune components or cells (17). For example, Complete Blood Count (CBC) and immunoglobulin levels are usually the initial tests performed in screening for suspected IEIs. Other accessible, useful screening tests for IEIs include peripheral blood smear (useful in diagnosing Chediak-Higashi syndrome), chest X-ray (helpful in identifying thymic hypoplasia in DiGeorge syndrome), decreased platelet number and size (suggestive of Wiskott-Aldrich syndrome), and serum calcium levels (DiGeorge syndrome). Functional assays typically include measuring T cell proliferation, neutrophil function, complement lysis, and vaccine responses (18). Additional tests, such as T cell receptor excision circles (TRECs), Kappa-deleting recombination excision circles (KRECs), and mutation analyses, are conducted in molecular laboratories (19). Through targeted investigations of defects in the immune pathways, IEIs detectable by a general physician include antibody deficiencies, which are among the most common forms of IEIs found mostly in children, and may also present in adults (17). The most common B-cell (antibody) defect is isolated IgA deficiency; other antibody deficiencies include Common variable immunodeficiency (CVID) and X-linked agammaglobulinemia (XLA); the most common T cell defect is DiGeorge syndrome (1). Chronic granulomatous disease (CGD) is the most well-known phagocyte defect, while the most common NK cell defect is X-linked lymphoproliferative syndrome and Primary hematophagocytic lymphohistiocytosis (HLH) (1).

IEIs are a neglected issue in Uganda, where infectious diseases are common and the main focus of healthcare. Uganda has a national patient organization, the Uganda Primary Immunodeficiencies Patient Organization (PIPO), which is a member of the International Patient Organization for Primary Immunodeficiencies (IPOPI). However, initiatives aimed at strengthening awareness, systematic screening, and diagnostic capacity for IEIs remain limited. As a result, IEIs are rarely considered in the differential diagnoses, and their diagnosis is often missed. Consequently, there are no nationwide data on the prevalence of IEIs in Uganda. Most importantly, the level of knowledge about IEIs is understudied in the country. This study was designed to assess graduate-level medical doctors’ understanding of the fundamental functions of immune system components and their ability to connect and relate this knowledge to clinical signs, warning and specific signs, as well as relevant immunological investigations related to IEIs. The findings will be used to establish a baseline understanding, which will support the IEIs-focused training.

## Method

### Study design, participants and data collection

This quantitative cross-sectional study was conducted between October 2023 and June 2024 at Makerere University College of Health Sciences, one of the constituent colleges of Makerere University. This university trains over 150 graduate-level Medical doctors annually in various graduate courses, including pediatrics, internal medicine, immunology, and clinical microbiology. Briefly, the sample size was calculated for a cross-sectional questionnaire assessing knowledge and clinical practice regarding Inborn Errors of Immunity (IEIs) among graduate-level medical doctors. Because the source population was finite (N = 150), we applied the single population proportion formula with finite population correction. Assuming a 95% confidence level (Z = 1.96), a margin of error of 5%, and a conservative estimated proportion of 50% (given the absence of prior local data), the initial sample size for an infinite population (n_0_ = 384) was adjusted using the finite population correction formula, resulting in a minimum required sample of 108 participants. To account for an anticipated 5% non-response rate, the final sample size was increased to 114 participants.

The participants included 114 Medical doctors pursuing a Master of Medicine in pediatrics, a Master of Medicine in internal medicine, and a Master of Science in immunology and clinical microbiology at Makerere University. These doctors work in different hospitals of the country and are knowledgeable about the workings and infrastructure of various health centers and hospitals in Uganda, making them a representative group for this study. Most regional referral hospitals and several private hospitals in Uganda have access to basic diagnostic tests relevant to IEI screening, including complete blood counts, chest X-rays, peripheral blood smears, platelet counts and size assessment, and serum calcium measurements. More advanced immunological investigations, such as flow cytometry and molecular testing, are available in approximately three specialized laboratories in Kampala. However, these facilities are primarily focused on infectious disease diagnostics and are not specifically structured, staffed, or trained for the routine screening and diagnosis of IEIs. All the 114 participants (100%) had prior training on the basic functions of different parts of the immune system; however, only 34 (30%) had prior introductory knowledge of IEIs.

A questionnaire adapted and modified from Hariyan et al. (4) was used. The tables included in the manuscript are representative of the questionnaire used in the study. The questionnaire of 42 questions was used to compare responses of 114 graduate medical doctors pursuing a Master’s of Medicine in pediatrics, Internal Medicine, and immunology and Clinical Microbiology. The questionnaire was divided into four sections: Basic functions of immune cells and clinical manifestations related to defects in B cell, T cell, phagocyte, complement, NK cell functions (10 questions); when to suspect IEIs [Warning signs in both children and adults (13 questions); specific signs about IEIs (4 questions); immunological investigations of suspected IEIs related to B cell, T cell, phagocyte, Complement, NK cell deficiencies (15 questions). The questions were of “True” (indicating correct knowledge) and “Not sure” (indicating incorrect or uncertain knowledge) type The survey was announced to potential participants on the day of the survey. The questionnaires were distributed on-site during class hours, and the graduate doctors completed them within 30 minutes. A paper version of the questionnaire was used.

### Data management and analysis

All questionnaires were checked for completeness and consistency before data entry. The data were coded and entered into Microsoft Excel and later exported to SPSS version (USA) for analysis. Descriptive statistics were used to summarize the data. Specifically, the proportions of correct and incorrect responses to each question related to basic functions of the different cells of the immune system; clinical manifestations related to defects in B cell, T cell, phagocyte, complement; when to suspect IEIs [Warning signs in both children and adults; specific signs about IEIs (XLA, SCID, CVID, CGD); immunological investigations of suspected IEIs related to B cell, T cell, phagocyte, Complement, and NK cell deficiencies were calculated and expressed as percentages. These percentages were utilized to assess graduate doctors’ overall knowledge about IEIs. All data were anonymized and stored securely to maintain participant confidentiality.

### Ethics statement

Ethical approval of the study was obtained from the Mulago Hospital Research and Ethics Committee (approval number: MHREC**-**2021-38). The study participants’ names were anonymized and cannot be linked back to the members of the graduate medical student trainees at Makerere University College of Health Sciences. Administrative clearance was obtained from the Uganda National Council for Science and Technology (approval number: HS1867ES). The participants provided their written informed consent to participate in the study.

## Results

The majority of the respondents were female: 64 (56.1.6%) versus male, 50 (43.9%).

### Knowledge of immune cell functions and clinical manifestations related to defects in the immune cells was high among graduate medical doctors

Recent studies indicate that a deeper comprehension of the basic roles of different immune cells and clinical manifestations related to defects in these cells and immune pathways improves the diagnosis and reporting of IEIs (20, 21). The knowledge concerning immune system functions and their relation to IEIs has not been assessed in Uganda. This segment of the survey aimed to evaluate 114 doctors’ grasp of the specific roles played by B cells, T cells, phagocytes, complement proteins, and natural killer cells, with responses categorized as “True” (indicating correct knowledge) and “Not sure” (indicating incorrect or uncertain knowledge). The results are summarized in **Table 1**. We found that 106 respondents (93%) correctly understood that B cells produce antibodies and are involved in opsonization and complement activation, with only 8 doctors (7%) expressing uncertainty. Regarding T cell function, 105 respondents (92.1%) correctly identified that T cells play a critical role in viral immunity and protection against intracellular pathogens, as well as having an immune regulatory function, with 9 doctors (7.9%) unsure. This high rate of correct responses reflects a solid grasp of T cell functions, especially in infections. Additionally, 110 respondents (96.5%) correctly acknowledged that phagocytes eliminate microbes and contribute to antigen presentation, with only 4 doctors (3.5%) uncertain. Meanwhile, 93 doctors (81.6%) accurately answered that complement proteins like C3b tag pathogens, marking them for phagocytosis by immune cells such as macrophages, with 21 doctors (18.4%) uncertain. This positive response indicates a good understanding of the role of complement in complement-mediated phagocytosis. 97 doctors (85.1%) correctly stated that natural killer cells destroy virally infected and tumor cells, with 17 doctors (14.9%) unsure. This moderate rate of correct responses suggests a sufficient understanding of NK cells’ critical role in innate antiviral and tumor immunity. Overall, the high rate of correct responses suggests a solid understanding of the basic functions of different immune cells.

**Table 1:** Comparison of the number and percentage of responses on basic immune cell functions and clinical manifestations related to defects in B cell, T cell, phagocyte, complement, NK cell functions.

No	Question	Responses (n=114)			
		True		Not sure	
		n	%	n	%
The following questions assess your knowledge of the basic functions of B cells, T cells, phagocytes, complement proteins and NK cells					
1	B cells make antibodies and have a role in opsonization and complement activation.	106	93	8	7
2	T cells are essential in viral immunity and immunity to intracellular pathogens and have an immune regulatory role.	105	92.1	9	7.9
3	Phagocytes clear microbes and play a role in antigen presentation	110	96.5	4	3.5
4	Complement proteins (e.g., C3b) coat pathogens, marking them for phagocytosis by immune cells like macrophages	93	81.6	21	18.4
5	Natural killer cells destroy virally infected cells and tumor cells	97	85.1	17	14.9

	The following questions assess your knowledge on clinical manifestations related to defects in B cells, T cells, phagocytes, complement proteins and NK cells	Responses (n=114)			
		Yes (true)		Not sure	
		n	%	n	%
6	B cell defects cause bacterial infections with predominantly capsulated microorganisms such as *H. influenza, Strepcoccus pneumoniae*	94	82.5	20	17.5
7	T-cell defects lead to opportunistic infections and autoimmunity.	78	68.4	36	31.6
8	Phagocyte (neutrophil) defects lead to bacterial infections and poor wound healing	82	71.9	32	28.1
9	Complement defects lead to bacterial infections and autoimmunity	77	67.5	37	32.5
10	Natural killer cell deficiency leads to viral susceptibility and malignancy	91	79.8	23	20.2

The next subsection of the survey aimed to evaluate the understanding of clinical manifestations related to defects in B-cell, T-cell, phagocyte, complement, and NK cell functions. We found that 94 doctors (82.5%) correctly understood that B cell defects result in bacterial infections predominantly caused by encapsulated microorganisms such as *H. influenzae* and *Streptococcus pneumoniae*, while 20 doctors (17.5%) were unsure. Regarding T cell function defects, 78 doctors (68.4%) accurately identified that T cell defects lead to opportunistic infections and autoimmunity, with 36 doctors (31.6%) uncertain. This average correct response rate reflects a moderate understanding of clinical manifestations associated with T cell defects. Additionally, 82 doctors (71.9%) properly recognized that phagocyte (neutrophil) defects lead to bacterial infections and poor wound healing, while 32 doctors (28.1%) were unsure. For complement defects, 77 doctors (67.5%) correctly answered that such defects cause bacterial infections and autoimmunity, with 37 doctors (32.5%) uncertain. This average response indicates a fair understanding of the manifestations of complement dysfunction. Finally, 91 doctors (79.8%) accurately indicated that NK cell deficiency leads to increased viral susceptibility and malignancy, with 23 doctors (20.2%) unsure.

### Low knowledge of warning signs of IEIs among graduate medical doctors

Adequate awareness of the warning signs of IEIs is essential for guiding timely investigation and early diagnosis of these conditions, which facilitates early supportive interventions such as antibody replacement, antibiotic prophylaxis, avoidance of live vaccines, and potentially curative hematopoietic stem cell transplantation (HSCT) (4). Recurrent infections can often be the first indication of IEIs (14, 15, 20). Therefore, a comprehensive history of infections, including the site, severity, occurrence of unusual organisms, and treatment response, is crucial. The understanding of warning signs of IEIs has yet to be assessed in Uganda. This part of the survey evaluated participants’ knowledge of common warning signs that should prompt a general medical practitioner to suspect IEIs. We utilized the warning signs established by the Jeffrey Modell Foundation Advisory Board and other organizations, with minor modifications (14, 15). A significantly higher percentage of doctors recognized that a child’s failure to gain weight or grow normally could indicate IEIs (76% versus 24%) and acknowledged the importance of a family history of IEIs (72% versus 28%). However, participants displayed less confidence regarding the remaining warning signs of IEIs, with correct identification rates ranging from 18% to 44%, revealing gaps in knowledge on these signs (**Table 2**).

**Table 2:** Comparison of the number and percentage of responses on warning signs of IEIs in adults and children.

No	Question	Responses (n=114)			
		Yes (True)		Not Sure	
		n	%	n	%
The following questions assess your knowledge of warning signs of IEIs					
1	Failure of an infant to gain weight or grow normally could be a sign of IEIs	87	76	27	24
2	Four or more new ear infections within one year could be a sign of IEIs	41	36	73	64
3	Two or more serious sinus infections within one year could be a sign of IEIs	34	30	80	70
4	Two or more months of antibiotics, with little effect, may be a warning sign of PIDs	37	32.5	77	62.5
5	Two or more pneumonias within one year could be a warning sign of IEIs	35	31	79	69
6	Recurrent deep skin or organ abscesses could be a warning of IEIs	32	28	82	72
7	Persistent thrush in the mouth or fungal infection on the skin could be a sign of IEIs	44	38.6	70	61.4
8	Recurrent need for intravenous antibiotics to clear infection could be a warning sign of IEIs	40	35.1	74	64.9
9	Two or more deep-seated infections including osteomyelitis, meningitis, abscesses, or sepsis may be a warning sign of IEIs	42	36.8	72	63.2
10	Autoimmune diseases and autoinflammation can be symptoms of IEIs	24	21.1	90	78.9
11	Certain malignancies and severe eczema in both children and adults can be a sign of IEIs	18	15.8	96	84.2
12	Invasive infection with normally inoffensive mycobacteria may be a warning sign of IEIs	24	21	90	79
13	A family history of primary immunodeficiency predicts IEIs	82	72	32	28

### Low graduate medical doctors’ knowledge of specific signs of IEIs

It is important to recognize how specific IEIs present and to relate this presentation to the pathophysiology of the disease in order to determine appropriate investigation and treatment. This part of the study examined the knowledge of graduate medical doctors regarding specific signs and symptoms of IEIs, with a particular focus on conditions that a junior doctor may encounter in an infectious disease clinic setting (1, 4). This included antibody deficiencies such as X-linked Agammaglobulinaemia (XLA) and Common Variable Immune Deficiencies (CVID); T cell defects, including Severe Combined Immunodeficiency; and phagocyte defects like Chronic Granulomatous Disease (CGD). Most participants felt less confident about recognizing the specific signs of these IEIs, with correct recognition rates ranging from only 13% to 30%, suggesting significant gaps in knowledge regarding the specific signs of IEIs (**Table 3**).

**Table 3:** Comparison of the number and percentage of responses on specific clinical manifestations of IEIs (XLA, SCID, CVID, CGD).

No	Question	Responses (n=114)			
		Yes (true)		Not sure	
		n	%	n	%
The following questions assess your knowledge of specific clinical manifestations of IEIs					
1	Recurrent sinopulmonary infections involving encapsulated bacteria in children suggest a defect in antibody production (B cell defect), XLA, or a combined cellular and humoral defect	26	23	88	77
2	A history of prolonged viral infections, opportunistic infections, autoimmunity, and failure to thrive (in the setting of an infant or child) suggests a possible T cell defect such as SCID.	30	26.3	84	73.7
3	Common Variable Immunodeficiency (CVID) is defined by low IgG plus low IgA and/or low IgM	13	11.4	101	88.6
4	Recurrent infections caused by fungi or bacteria can be a sign of Chronic Granulomatous Disease.	27	23.7	87	76.3

### Low knowledge and practice on immunological investigations for suspected IEIs related to B cell, T cell, phagocytes, complement, NK cell deficiencies

Adequate knowledge and practice of immunological investigations to explore various immune pathways related to the pathophysiology and mechanisms underpinning IEIs guides the establishment of laboratory infrastructure focused on these disorders and the selection of appropriate tests for early diagnosis (22, 23). There are both quantitative and functional assays (22). The complete blood count (CBC) of white blood cells is an essential test, as it reveals any severe blood abnormalities that may arise from IEIs. For example, patients with severe combined immunodeficiency (SCID), one of the most serious IEIs, typically exhibit very low levels of lymphocytes (22). In Uganda, most doctors have access to CBC testing; however, knowledge regarding the practice of immunological investigations related to IEIs diagnosis is not known. The study examined the awareness of graduate medical doctors about IEIs -focused immunological investigations, including CBC and tests focused on B cells, T cells, phagocytes, complement, and natural killer cell defects. The participants displayed limited knowledge and utilization of all the immunological investigations for IEIs that were included in the assessment, with correct response rates ranging from 0% to 6% (**Table 4**). The vast majority (over 90% in all cases) had neither studied, requested, referred, nor performed any of the listed tests, with particularly notable findings for specialized tests such as measuring switched memory B cells and conducting genetic testing. Newborn screening using T-cell receptor excision circles (TRECs) and Kappa-deleting recombination excision circles (KRECs), NK cell assays, and complement assays revealed that no participants (0%) reported any experience. Even for more general tests like complete blood count and differential counts of various white blood cells, only a small minority (6%) had requested them to screen for IEIs. These findings suggest that graduate medical doctors in Uganda possess very limited knowledge regarding IEIs immunological investigations.

**Table 4:** Comparison of the number and percentage of responses on immunological investigations for suspected IEIs related to B cell, T cell, phagocytes, complement, NK cell deficiencies.

No	Question	Responses (n=114)			
		Yes		Not sure	
		n	%	n	%
The following questions assess whether you have ever studied, requested, or performed laboratory tests listed below in your practice to screen for or diagnose suspected IEIs as related to defects in B cells, T cells, NK cells, phagocytic cells, complement system.					
1	Complete blood Count (CBC) and differential counts of the various white blood cells	6	5.3	108	94.7
Investigations for screening B cell defects					
2	Measurement of Immunoglobulins- IgG, IgA, IgE, IgM levels in blood	2	1.8	112	98.2
3	B cell immunophenotyping (CD19, CD20), CD40/CD40L expression by flow cytometry analysis	3	2.6	111	97.4
4	Measurement of switched memory B cells	0	0	114	100.0
5	Genetic testing (DNA sequencing) (e.g. BTK)	0	0	114	100.0
Investigations for screening T cell defects					
6	T cell immunophenotyping (e.g. CD3, CD4. CD8, CD40/CD40L expression) by flow cytometry analysis	1	0.9	113	99.1
7	T cell subpopulations, and functional (proliferation) tests in response to mitogen such as PHA	1	1.4	113	98.6
8	Newborn screening using T-cell Receptor Excision Circle (TRECs), and Kappa-deleting Recombination Excision circle (KRECs)	0	0	114	100.0
9	Genetic testing (DNA sequencing, e.g. IL-2RG, RAG1/2)	0	0	114	100.0
Investigations for screening NK cell defects					
10	NK cell immunophenotyping (CD3, CD16, CD56)	1	1.4	113	98.6
11	NK cell cytotoxicity assays	0	0	114	100.0
Investigations for screening Phagocyte defects					
12	Granulocyte function tests (e.g., DHR oxidative burst test)	3	2.6	111	97.4
13	Phagocyte cell (monocyte, neutrophil) immunophenotyping	4	1.8	112	98.2
Investigations for screening Complement defects					
14	CH50 assay	0	0	114	100.0
15	AH50 assay	0	0	114	100.0

## Discussion

A strong foundational knowledge of the immune system, its components, cells, functions, and clinical manifestations-related to defects in individual immune pathways is essential for doctors to recognize and manage a wide range of health conditions (4, 5). This is especially critical in the management of Primary Immunodeficiency, where immune dysregulation can present with subtle warning signs, specific clinical manifestations, and measurable immunological changes. Doctors must be equipped to understand immune system mechanisms and apply this knowledge to detect and diagnose immune-related changes associated with primary immunodeficiency. This study assessed graduate medical doctors’ understanding of the fundamental functions of immune cells, and their ability to relate this knowledge to clinical manifestations related to defects in these cells, warning and specific signs, and immunological investigations relevant to IEIs.

The findings revealed a clear divide: while participants demonstrated a solid understanding of immune cells’ function, their awareness of clinical warning signs, specific signs, and immunological investigations associated with IEIs was significantly limited. This suggests that although theoretical knowledge of immunology exists, its translation into practice, especially in the domain of IEIs, remains underdeveloped. This limitation may be due to insufficient education or hands-on training in immunological investigations related to IEIs diagnosis.

This gap may be attributed to several factors, including insufficient emphasis on IEIs-related pathophysiology and immunopathology in medical education, a lack of sensitization regarding both immunological investigations and laboratory infrastructure for IEIs diagnosis, and an overemphasis on the diagnosis and management of infectious diseases in this setting. These findings highlight the urgent need for targeted training that connects clinical immunology science with real-hospital clinical scenarios involving IEIs, enabling doctors to recognize immune-related signs specific to IEIs.

In agreement with prior research, our findings confirm that while most physicians possess a strong theoretical understanding of immune system functions, they may often lack confidence in applying this knowledge to complex clinical presentations, such as those seen in IEIs (5, 24, 25). In fact, a previous survey of family physicians revealed a low awareness of IEIs guidelines (only 4% aware) compared to Immunologists (79%), with differences in diagnostic use and management, suggesting a need for broader IEIs -focused training (24). Previous studies have similarly reported that medical practitioners demonstrate limited awareness of the immunological consequences associated with IEIs, possibly due to the underrepresentation of IEIs immunopathology in standard medical curricula (24, 26–28). A study conducted in Brazil found that 77% of physicians were unfamiliar with IEIs warning signs, and only 18. 3% of pediatricians answered clinical scenario questions correctly (24). Moreover, Brazilian physicians highlighted that limited exposure to IEIs during training contributed to low recognition of IEIs warning signs and diagnostic delays (23). A study in Turkey revealed that 33% of physicians reported no prior IEIs training, and only 6.6% recognized all specific and diagnostic warning signs (29). Turkish researchers emphasized that the lack of formal training programs perpetuates low awareness, as evidenced by physicians’ reliance on outdated diagnostic practices. Furthermore, a study in Ukraine revealed that Ukrainian medical students averaged 59. 2% correct answers on IEIs knowledge tests, with particularly low scores for adult presentations and IEIs subtypes (26). The Ukrainian study explicitly identified insufficient education in medical curricula, noting that students struggled most with adult IEIs manifestations and specific immunopathological mechanisms.

In contrast to earlier studies suggesting that increasing physician familiarity with immunological diagnostics in broader clinical settings (e.g., infectious diseases and autoimmune disorders) leads to better clinical application in IEIs diagnosis our findings suggest that this trend has not extended to the context of IEIs, at least in our setting (30). This discrepancy may be explained by the fact that immunological changes related to IEIs are nuanced, less characterized, and often overshadowed by infectious diseases and cancers, making them harder to detect without specialized and IEIs - focused training (28).

One of the key strengths of this study lies in its focused assessment of graduate medical doctors’ knowledge across both immunological theory and its clinical application to IEIs, a topic that remains underexplored in Uganda where infectious diseases are the main focus of diagnosis. Additionally, the inclusion of doctors from diverse clinical backgrounds and hospitals enhances the generalizability of the findings across multiple specialties and healthcare settings.

This study has several limitations that should be acknowledged. First, the study included only graduate-level medical trainees as respondents, leaving out qualified and experienced pediatricians, Internists or Immunologists, and may not represent the broader physician population IEIs awareness. Second, the assessment relied, in part, on self-reported knowledge, which may not fully capture doctors’ actual clinical performance.

Future studies and activities should focus on strengthening clinical immunology knowledge in medical training regarding clinical manifestations and Laboratory investigations of IEIs: Immunology curriculum development should include targeted content on pathophysiology and mechanisms of IEIs, general warning and specific signs, and relevant IEIs -focused immunological and genetic investigations. Case-based learning should focus on identifying IEIs specific signs and interpreting pertinent laboratory results.

In summary, there is limited knowledge on warning, specific signs and immunological investigations for IEIs among graduate-level medical doctors in Uganda. Enhanced training is urgently needed to improve IEIs recognition and diagnosis. Thus, the findings establish a baseline understanding that will support IEIs -focused training of medical students, improve research, diagnosis and reporting of IEIs in Uganda.

## Data Availability

The raw data supporting the conclusions of this article will be made available by the authors, without undue reservation.
